# Effects of a Participatory School-Based Intervention on Students' Health-Related Knowledge and Understanding

**DOI:** 10.3389/fpubh.2020.00122

**Published:** 2020-04-24

**Authors:** Helmut Strobl, Katharina Ptack, Clemens Töpfer, Ralf Sygusch, Susanne Tittlbach

**Affiliations:** ^1^Institute of Sport Science, University of Bayreuth, Bayreuth, Germany; ^2^Department of Sport Science and Sport, Friedrich-Alexander-University Erlangen-Nürnberg (FAU), Erlangen, Germany

**Keywords:** physical literacy, physical activity (exercise), secondary school, physical education (P.E.), questionnaire, Health.edu

## Abstract

**Introduction:** The development of knowledge and understanding in relation to movement and health is a basic requirement to facilitate lifelong engagement in physical activity with its accompanying possible health benefits. To train teachers in applying adequate strategies, implementation studies have often shown little acceptance of traditional top-down approaches. Thus, the purpose of the Health.edu project was to develop, implement and evaluate effective and feasible measures addressing students' health-related knowledge and understanding (HKU) in physical education (PE) via a participatory approach.

**Materials and Methods:** For evaluation, a controlled pre-post-test study with 233 students from eight different secondary schools in Germany was carried out. Four schools (with two PE teachers at each school) comprised the intervention group and they participated in a 1-year participatory planning process to conceptualize and implement evidence-based PE lessons addressing students' HKU. Control schools carried out their regular PE lessons. Evaluation followed a mixed-methods research design, assessing program implementation via written documentary technique as well as program effectiveness using a standardized questionnaire.

**Results:** Results show a significant intervention effect on students' HKU with a medium effect size. However, due to the participatory process, there were considerable differences between the intervention schools that were involved. Student's HKU improved most in schools where program implementation corresponded to relevant principles of fostering HKU.

**Discussion:** The present study purposefully dispensed with any structured intervention programs for PE teachers to follow. The results show the potential effects of this participatory approach to strengthen student's HKU. However, the participatory planning does not always work in the intended manner, emphasizing that numerous contextual factors influence the implementation process.

## Introduction

For all individuals, health is a resource that needs to be maintained and protected. Therefore, against the background of a salutogenic and holistic approach, people should be enabled to increase control over, and to promote, their health (1). This emphasis is the result of an increasing public awareness of health problems such as obesity, cardiovascular diseases, and mental health (2). To comply salutogenic and holistic requirements, concepts of health promotion should focus on salutary rather than risk factors, and always see the entire person rather than the single disease (3). Schools especially are regarded to be ideal settings for health promotion, due to the easy accessibility to children and adolescents as well as the large amount of time students spend at school (4, 5). Thus, health promotion at school must be linked directly to school educational development and school's educational goals (6, 7). Considering physical education (PE), there is currently a broad international discussion about how to link the educational aspects of physical activity and physical activity promotion in current PE curricula (7–11). To achieve the wide range of possible educational outcomes of PE for school-age children, a focus on health outcomes through fitness training, without any reflection concerning educational purposes, is not sufficient. Instead, the overall aim should be to provide young people with the knowledge, skills and understanding necessary to perform various physical activities and maintain healthy lifestyles (9, 12–16). This is a necessary precondition for many health enhancing effects (17) and thus also important from a public health point of view (18).

The notion of physical literacy (PL) provides helpful pedagogical orientation for PE, as it captures the essence of what a quality PE aims to achieve (13, 19, 20). PL is defined as “the motivation, confidence, physical competence, knowledge and understanding to value and take responsibility for engagement in physical activity for life” (21). The concept was introduced by Whitehead (22) and since then PL has gained a growing scholarly interest (13, 19, 23, 24). As definitions adopted from different organizations and research groups differ, Edwards et al. provided a systematic review of core attributes of PL (25). Affective and cognitive properties, as well as physical capabilities emerged as particularly common subthemes of the PL construct. Being physically literate is conceived to be the result of the integrated interaction of these domains (20, 25). PL is further considered a basic requirement to facilitate lifelong engagement in physical activity with its accompanying possible health benefits (13, 23, 26, 27). Focusing on the relationship of the different PL domains, some authors denote the cognitive attributes of knowledge and understanding as fundamental to achieving the other domains and the overall concept of PL (23, 28, 29). In that sense, students should acquire knowledge and understanding in relation to movement and health. This includes for instance a profound knowledge and understanding of the principles of movement and performance, but also of the requirements, antecedents and values of participating in a physically active lifestyle (25, 27, 28).

The concept of PL including its cognitive attributes of knowledge and understanding is also widely accepted in German PE curricula^1^ and pedagogical concepts (14, 30, 31). In line with the international discussion, students should be enabled to care for their own health and not to produce primarily direct health outcomes (14). The aim is to develop students' knowledge and understanding to practice sport in a healthy way, to assess the health effects of one's own sport activities and, if necessary, to modify the activity in order to be more healthy (32). Thus, the development of knowledge and understanding in relation to health to facilitate students making healthy choices for physical activity (33) is in particular a major concern in German PE.

In order to target health-related knowledge and understanding (HKU) in PE, there is a need for student-centered teaching-learning processes, combining delivery of health-related knowledge with experiences through physical activity (13, 23, 34–37). However, research indicates PE teachers' limited knowledge in defining appropriate learning outcomes for HKU and how to deliver those aspects within their PE lessons (8, 28, 34, 35, 38). To train teachers in applying adequate strategies for delivering health-related knowledge, implementation studies have often shown little acceptance of traditional top-down approaches (36, 37). Instead, there is evidence for the potential of a participatory approach (39). This includes the collaboration of relevant members of a setting (e.g., teachers, principal, and students at school) with a scientific research team in order to plan, implement and evaluate health-promoting measures. The corresponding communication and interaction processes can enhance knowledge and competencies concerning evidence-based and feasible health-promoting measures among all involved stakeholders (40, 41). Furthermore, equal participation of all stakeholders in discussions and decision-making may enhance identification of all participants with the actions taken and increases chances of sustainable implementation (42, 43). For those reasons, the Health.edu project strives to develop, implement and evaluate effective and feasible student-centered measures for the enhancement of HKU in PE via a participatory approach^2^. The focus of this paper is mainly to report the evaluation of the project's effectiveness, i.e., change of students' HKU due to the intervention. The overall research question is whether it is possible to strengthen students' HKU via a school-based participatory approach.

## Materials and Methods

### Design

We carried out a quasi-experimental pre-post-test study design over one academic year with eight secondary schools (four intervention schools and four control schools), evaluating program implementation as well as program effectiveness (44, 45). Evaluation followed a mixed methods design. The effectiveness of the intervention was assessed by a standardized paper and pencil questionnaire. However, as differences between intervention schools may occur due to the participatory process, we also took the evaluation of program implementation via qualitative methodology into account. Therefore, written documentary technique was used additionally to document and analyses the processes throughout the intervention.

### Intervention

The goal of the intervention was to enable students to make healthy choices regarding physical activity by strengthening their HKU. To match scientific evidence concerning delivery of health-related knowledge in PE with actual prerequisites within schools, an effective way of exchanging knowledge between scientists and school stakeholders (e.g., principals, students, and PE teachers) is necessary. Therefore, we used a participatory approach since this has been shown to be effective in previous studies concerning health promotion (46–48). To ensure knowledge exchange processes, we facilitated so-called cooperative planning groups (consisting of scientists, students, principal and PE teachers) at each of the four intervention schools. Planning groups were maintained throughout the academic year. The schedule of the single planning group meetings (see Table 1) was similar at each school.

**Table 1:** Schedule of the single planning group meetings.

**Meeting**	**Agenda**
1	• Getting to know each other. • Scientific input on salutogenic and holistic health promotion. • Participatory development of a common salutogenic and holistic understanding of health and fitness among all stakeholders involved. • First considerations on health-related learning outcomes for PE.
2	• Participatory definition of health-related learning outcomes for the single PE classes involved. • Scientific input on student-centered teaching strategies to deliver health-related knowledge and understanding in PE: cognitive activation, reflection, relevance to everyday life, collaborative learning. • First considerations on adequate student-centered teaching strategies and their implementation in PE, according to the defined learning outcomes.
3	• Participatory selection of student-centered teaching strategies to deliver health-related knowledge for the single PE classes involved. • Participatory development of an implementation schedule for the selected student-centered teaching strategies within the next PE classes.
4	• Exchange of experiences on the implementation of the selected student-centered teaching strategies in the single PE classes involved. • Participatory discussion of improvement strategies. • First considerations on ensuring sustainability of the initiated processes within the single schools involved.
5	• Exchange of experiences on the implementation of the selected student-centered teaching strategies in the single PE classes involved. • Definition of structures and responsibilities for ensuring sustainability of the initiated processes within the single schools involved.

In the first two meetings, participants of planning groups discussed and defined health-related learning-outcomes for their PE lessons. Learning outcomes should follow a holistic understanding of health and fitness, integrating physical as well as psychosocial aspects (14, 28, 33). On the one hand, students should learn how to apply the short- and long-term benefits of physical activity regarding improvement of physical fitness at school as well as in their spare time. They also should gain knowledge and understanding about their activity-related behavior in terms of risk factors, injuries and illnesses. On the other hand, PE should improve students' knowledge and understanding of how social and mental well-being are interrelated with physical activity and how personal well-being can be developed by means of physical activity.

In the second meeting, the research group additionally provided scientific input on student-centered teaching strategies (cognitive activation, reflection, relevance to everyday life, collaborative learning). Research shows, that the application of student-centered teaching-strategies during PE lessons best supports students' cognitive processes of accessing, understanding and appraising health-related information (8, 49, 50). “Cognitive activation” means to ask students for previous activity-related experiences and link it to new content (e.g., asking students for known warm-up exercises and discussing their relevance to the subsequent sport activities). “Reflection” refers to providing students with stimuli, which let them think about their experiences following the pursued activities (e.g., reflecting the effects of a High-Intensity-Training on heart rate and subjective exhaustion or reflecting the effects of different stretching exercises on mental well-being). “Relevance of everyday life” means to consider topics in PE lessons that are relevant for the students (such as fitness training for a forthcoming ski course). “Collaborative learning” finally refers to giving the students a task, which has to be solved by working together (e.g., providing sheets with information on a muscle building training circuit and instructing the students to organize single circuit stations).

The participants of the planning groups jointly selected in meetings two and three adequate student-centered teaching strategies for the previous defined learning outcomes and discussed their implementation within the next PE classes. In the discussions, great importance was attached to ensure that the tasks set by PE teachers are always combined with experiences through physical activity. That is, teachers should not only deliver health-related knowledge theoretically, but also combine it directly with physical experiences (e.g., different kinds of running intensity and its effects on heart pulse in combination with pulse monitoring or different methods of muscle stretching and its effects on relaxation).

Finally, PE teachers implemented these conceptualized lessons in their PE classes and afterwards a discussion of their experiences took place among all participants of the planning groups. The stakeholders involved also discussed how to ensure sustainability of the initiated processes with the single schools concerned (meetings four and five).

### Participants

Recruitment of eight secondary schools occurred by contacting school principals via email, phone, or in-person. To account for similar socio-economic characteristics, schools were recruited in the same administrative district in Bavaria, Germany. To account for different cognitive abilities of the students, four secondary modern schools and four grammar schools were part of the study^3^. After agreeing to participate in the study, schools were placed–according to their willingness–into either the control or the intervention group (two grammar and two secondary modern schools, respectively). In each intervention school, two PE teachers (one female and one male respectively) participated with their corresponding PE classes voluntarily in the study. The total pre-post-sample of the students included 233 students with the age range of 11–17 years (*M* = 14.66, *SD* = 1.27). Table 2 provides an overview of participants pertaining to either intervention or control group.

**Table 2:** Number of students pertaining to intervention or control group.

	**Intervention**	**Control**	**Total**
Grammar school	70	43	113
Secondary modern school	71	49	120
Female	70	58	128
Male	71	34	105
7th grade	15	7	22
8th grade	25	8	33
9th grade	15	43	58
10th grade	86	34	120
Total	141	92	233

### Measures

Regarding the evaluation of program implementation, protocols of each planning group meeting were compiled (in total 19 protocols). An assistant documented relevant statements and resolutions over the course of the meeting. The assistant was not directly involved in the research project, warranting a “neutral” view on the discussions. At the end, all participants of the planning group had the opportunity to add their most important findings throughout the discussions. The protocols allow an analysis of how a holistic understanding of health and student-centered teaching methods were addressed within the PE lessons.

**Figure 1:**
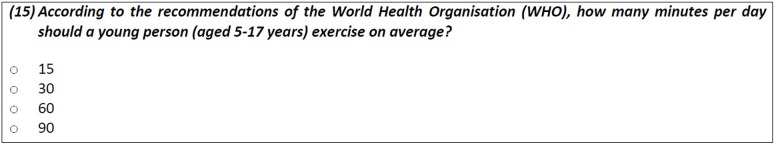
Example for a single choice item of the standardized paper and pencil test.

**Figure 2:**
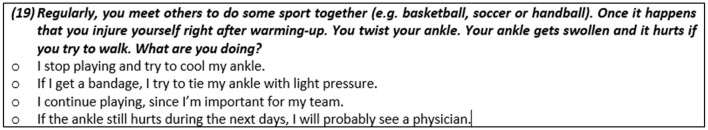
Example for a multiple choice item of the standardized paper and pencil test.

Concerning the evaluation of program effectiveness, the HKU of the students was measured at the beginning (*t*_0_) and the end (*t*_1_) of the school year through a standardized paper and pencil test. As there is a lack of an accurate empirical assessment of the outcomes of implemented teaching learning processes (23, 26), we used a well-developed and well-validated questionnaire (for further information on the validation process see next section 2.5) (33). The questionnaire consisted of 25 items including three different item formats: short text (8), single choice (10) and multiple choice (7). The questions follow a holistic understanding of health and fitness, integrating physical as well as psychosocial aspects, and are derived from current requirements in German PE curricula. One short text item was, for example, to describe a way of measuring heart rate without a pulse monitor. A second item was to name different perceived reactions of the body, while executing relaxation techniques. Figures 1, 2 give further examples for single choice and multiple-choice items.

Test instructors followed a predefined procedure (33). As the test is designed to be a power test and not a speed test, students had enough time to answer the questions: students were given 30 min to fill in the questionnaire. Item responses were assessed according to a coding guideline (33). Answers to the short text items needed to be rated on a four-step ordinal rating scale (0–3). Single and multiple-choice items were rated dichotomously (0–1). The inter-rater reliability (Kendalls Tau-c) proves a good or even very good level of congruence within the coding process (τ = 0,813). Missing data was treated as wrong answers (0) linking back to the fact that the person's individual HKU-level may be on a rather low level.

### Data Analysis

Qualitative data was analyzed via thematic content analysis (51–53) using MaxQDA12 software. In order to guarantee rigor and quality of the data analysis, attention was paid to the fact that the evaluation process is designed to be inter-subjectively comprehensive by documenting the process in detail (including the description of the theoretical grasp, the intervention, the context and the methodological approach) (54). At the beginning of the analysis, the data from the documentary techniques was coded according to the main categories “understanding of health” and “student-centered approaches.” The coding guide is successively generated by several rework loops and revisions with regard to definitions, anchor examples and coding rules. After grouping the codes to the corresponding main categories, another researcher encoded ~25% of randomly selected contributions to estimate intercoder–reliability (intercoder-reliability of 78%). Afterwards, all research members of the Health.edu project discussed and revised the coding guide. Intercoder–reliability subsequently increased up to 93%.

For the analysis of the quantitative data, values reflecting students' HKU were estimated upon the responsive behavior of the students using the Rasch Model (Partial Credit Model). Those values concern the probability that the students that were involved may correctly solve the questionnaire tasks. The applicability of the Rasch Model (Partial Credit Model) was successfully proved in a previous study (LR-test: Chi^2^ = 50.81; *df* = 40; *p* = 0.118) (33). The test exhibited an acceptable level of reliability (EAP/PV = 0.79). All items showed good values for item fit based on Weighted Mean Square (WMNSQ) within the range 0.8–1.2. Values for student's HKU were finally transformed into standard values (*M* = 500, *SD* = 100). Descriptive statistics for changes of student's HKU between *t*_0_ and *t*_1_ were calculated. An analysis of covariance was conducted to evaluate the treatment effect of the intervention on student's HKU at the end of the school year (HKU_t_1_) in contrast to the control group whilst adjusting for the effects of the educational level (i.e., type of school), gender and age. Those co-variates were chosen due their established association with literacy levels (55, 56). People with lower educational levels, older age and male gender tend to have lower health literacy levels. Among several social determinants of health literacy, education represents a very strong predictor, followed by age and gender (56). Additionally, students' HKU at the beginning of the school year (HKU_t_o_) was integrated as a covariate. By doing this, each student's score after the intervention is adjusted to his or her baseline score, which provides the advantage of being unaffected by baseline differences (57). Effect sizes (i.e., partial η^2^) were calculated for the F statistics. Statistical assumptions for ANCOVA were examined according to Field (58) and showed no major problems with kurtosis, skewness or homogeneity of variance.

The calculation of changes in students' HKU between pre- and post-test for each school of the intervention group and their corresponding 95% confidence intervals serve to illustrate the changes that occurred in every school. Effect sizes (i.e., Cohen's d) were calculated for mean differences. To consider dependency of the pre-post-data, calculation of **d** was based on mean differences between HKU_t_0_ and HKU_t_1_, divided by the standard deviation of HKU_t_0_, which was corrected by the correlation between pre- and post-test data (59). All quantitative data analyses were completed using R 3.3.0 and SPSS 25. Significance was set a priori at an alpha-value of < 0.05.

## Results

### Evaluation of Program Implementation

Altogether, 10 teaching units were developed and implemented in the four school specific planning groups (schools A, B, C, and D; Table 3). The teaching units showed differences in relation to the understanding of health. The majority of teaching units exclusively considered physical aspects of health (units 2, 3, 5, 6, and 8), whereby the other units (units 1, 5, 7, 9, and 10)–at least partly–also addressed psychosocial aspects of health. There were further substantial differences concerning student-centered approaches. Teaching units of schools A and B (units 1–5) were highly student-centered, as they considered all criteria such as cognitive activation, reflection, relevance to everyday life, and collaborative learning. They also consistently combined delivery of health-related knowledge with physical experiences. PE teachers of school D (units 8–10) neglected reflection and collaborative learning. However, as well as teachers of schools A and B, they prioritized educational aspects in contrast to direct health outcomes, attached great importance to cognitive activation via an intensive knowledge transfer and combined delivery of health-related knowledge with physical experiences. In contrast, teaching units of school C (units 6 and 7) only showed slight tendencies to consider cognitive activation and did not establish a link between delivery of health-related knowledge and physical experiences.

**Table 3:** Developed and implemented measures within PE in intervention schools (schools A, B, C, and D), including consideration of student-centered teaching strategies.

**School**	**Teaching unit**	**Description**	**Teaching strategies**
A	1	Female students from 10th grade chose from a given range of health-related themes (e.g., endurance training, anatomy and functions of the abdominal musculature, load intensity control, relaxation techniques, and basics of nutrition) and worked out contents independently. The PE teacher acted as supervisor, if necessary. Over the course of several lessons, the student groups presented theoretical background of the chosen themes in combination with practical exercises to their classmates.	CA, R, RL (partly), CL
	2	Male students from 7th grade passed a fitness test at the beginning, in the middle and at the end of the school year. In the meantime, they underwent a fitness training during PE lessons as well as part of their “homework.” The exercises addressed specifically muscles required for skiing (as preparation for the forthcoming school skiing course). Students voluntarily documented their training with a “movement log.” In the second half of the school year, students regularly reflected their training within PE lessons with the PE teacher and chose different exercises, if appropriate.	CA, R, RL, CL
	3	Volunteer male students from 7th grade worked out and conducted a sport-specific warm-up for PE independently. Warm-up should be based on experiences from their leisure sport activities as well as predefined criteria from the PE teacher concerning time for strengthening and stretching exercises. At the end of the lesson, the PE teacher reflected the warm-up with the whole class, emphasizing the potential health-enhancing or health-threatening effects of the chosen exercises.	CA, R, RL, CL
B	4	Volunteer male students from 10th grade worked out and conducted, supervised by their PE teacher, a lesson targeting strength training with one's own body weight. The exercises addressed specifically muscles required for swimming (as preparation for the forthcoming swimming class). For preparation, students had to research strength-training exercises for different swimming styles and to present the results to their classmates. They further worked out summaries of their results and provided them to other PE teachers of the school for further use.	CA, R, RL, CL
	5	The PE teacher worked out and conducted several lessons targeting dietary behavior and calorie expenditure through physical activity, as initiated by her female students from 9th grade. The students documented their physical activity via a smartphone app and assessed their calorie intake and expenditure by means of a provided worksheet. Additionally, they reflected the results among each other while executing a low-intensity walk. Moreover, the PE teacher provided information regarding recommendations for health-enhancing physical activity and nutrition while executing strengthening and stretching activities with her students.	CA, R, RL
C	6	Male and female students from 10th grade passed a fitness test in the middle of the school year. Students were encouraged to self-evaluate their fitness level and to prepare themselves for a second fitness test independently. However, PE teachers did not supervise the trainings activities of their students and the second fitness test did never take place.	CA (partly)
	7	Male and female students from 10th grade answered a questionnaire containing questions regarding their physical activity behavior and reasons for sedentary behavior. However, PE teachers did not discuss the results with their students.	CA (partly)
D	8	The PE teacher worked out and conducted several lessons targeting strength and endurance training with the use of dumbbells, medicine balls and one's own body weight, as initiated by his male students from 8th grade. The lessons were based on available literature addressing health and fitness training in PE. Additionally, he conducted a high-intensity-interval-training with his students. The PE teacher took an active part during the lessons to act as role model for his students. Furthermore, he emphasized delivery of knowledge regarding fitness training during the lessons.	CA, RL
	9	The PE teacher worked out and conducted a volleyball-specific warm up for her female students from 8th grade. Therefore, she chose exercises addressing strength endurance by means of a volleyball. The exercises were executed as part of a circuit-training course. At the end of the lesson, the PE teacher instructed their students how to perform a relaxation exercise. Delivery of knowledge regarding warm up and relaxation was an important part during the lessons.	CA, RL (partly)
	10	The PE teacher worked out and conducted a lesson targeting perception of balance/imbalance and health-promoting stretching of different muscles for her female students from 8th grade. Emphasize was on delivery of knowledge regarding stretching.	CA, RL (partly)

*CA, Cognitive activation; R, Reflection; RL, Relevance to everyday life; CL, Collaborative learning*.

### Evaluation of Program Effectiveness

According to Table 4, students' HKU generally improved across group, type of school, grade and gender. In detail, students from intervention schools (Δ55) improved their HKU more than students from control schools (Δ26). Greater improvements in HKU are also apparent in students from grammar schools (Δ60) in comparison to secondary modern schools (Δ29) and females (Δ48) in comparison to males (Δ38). Finally, students from lower grades realized greater HKU-improvements than students from higher grades (7th grade Δ58, 8th grade Δ52, 9th grade Δ48, 10th grade Δ37).

**Table 4:** Descriptive statistics (*x, SD*, and Δ for mean differences) for student's health-related knowledge and understanding at the beginning (HKU_t_0_) and at the end of the school year (HKU_t_1_) across group, type of school, grade, and gender.

	**HKU_t_**0**_**	**HKU_t_**1**_**	**Δ**
	**x (SD)**	**x (SD)**	
Intervention group	486 (94)	541 (109)	55
Control group	466 (85)	492 (89)	26
Grammar school	517 (81)	577 (98)	60
Secondary modern	441 (84)	470 (80)	29
7th grade	402 (78)	460 (101)	58
8th grade	421 (67)	473 (77)	52
9th grade	480 (87)	528 (100)	48
10th grade	507 (86)	544 (105)	37
Female	496 (88)	544 (100)	48
Male	457 (90)	495 (104)	38

Table 5 shows the results of the ANCOVA. Except for age (*F*_(1, 227)_ = 0.169, *p* = 0.692, η^2^ = 0.001), there is a significant relation to HKU_t1 for all covariates: HKU_t_0_ (*F*_(1, 227)_ = 134.250, *p* < 0.001, η^2^ = 0.372), type of school (*F*_(1, 227)_ = 26.840, *p* < 0.001, η^2^ = 0.106) and gender (*F*_(1, 227)_ = 4.414, *p* = 0.037, η^2^ = 0.019). While adjusting for the covariates, students in intervention schools had a significantly greater score of HKU_t_1_ in contrast to the control group (*F*_(1, 227)_ = 16.046, *p* < 0.001, η^2^ = 0.066; medium effect).

**Table 5:** Analysis of covariance (dependent variable: HKU_t_1_) with the factor intervention vs. control group and the covariates HKU_t_0_, type of school, age, and gender.

	**df**	**F**	**p**	**Partial η^**2**^**
**Covariates**				
HKU_t_0_	1, 227	134.250	0.000	0.372
Type of school	1, 227	26.840	0.000	0.106
Age	1, 227	0.169	0.682	0.001
Gender	1, 227	4.414	0.037	0.019
**Factor**				
IG vs. CG	1, 227	16.046	0.000	0.066

**Figure 3:**
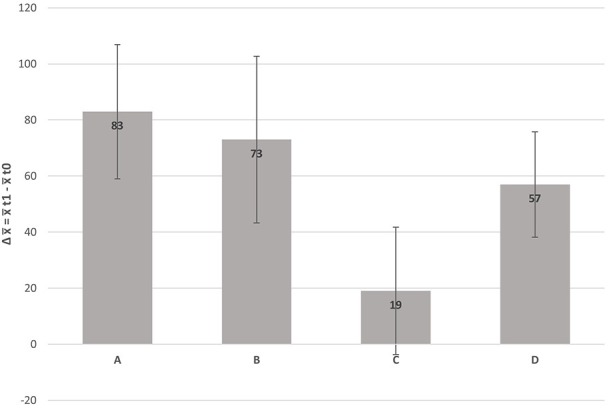
Average change (Δ) of student's health-related knowledge and understanding (HKU) between the beginning (*t*_0_) and the end of the school year (*t*_1_) and corresponding 95% confidence intervals, separated for each intervention school.

Results of the intervention differ considerably between schools (see Figure 3, Table 6). Significant mean differences with strong effect sizes of pre- and post-test data for students' HKU are apparent in schools A (*d* = 1.3), B (*d* = 1.0), and D (*d* = 1.3), but not for school C (non-significant mean difference with a small effect size, *d* = 0.2).

**Table 6:** Descriptive statistics (*x, SD*, and Δ for Mean Differences) for student's health-related knowledge and understanding at the beginning (HKU_t_0_) and at the end of the school year (HKU_t_1_) across intervention schools with adjusted Cohen's *d*.

	**HKU_t_**0**_**	**HKU_t_**1**_**	**Δ**	**d**
	**x (SD)**	**x (SD)**		
School A	493 (105)	576 (121)	83	1.3
School B	543 (78)	616 (96)	73	1.0
School C	479 (81)	498 (77)	19	0.2
School D	413 (66)	470 (73)	57	1.3

## Discussion

Health.edu strives to develop, implement and evaluate effective and feasible measures addressing health-related knowledge and understanding (HKU) in physical education (PE) via a participatory approach. The current paper investigates whether strengthening students' HKU via participatory developed and implemented PE lessons is possible. In total, 233 students from eight different secondary schools took part in the study. Four schools (with two PE teachers at each school) comprised the intervention group and they participated in a 1-year participatory planning process to conceptualize and implement various PE lessons. PE teachers in the planning groups were advised to conceptualize their PE lessons in order to develop students' health-related knowledge and understanding. Meanwhile, the control schools carried out their regular PE lessons. Evaluation followed a mixed-methods approach, assessing program implementation as well as program effectiveness.

In relation to program effectiveness, results showed a significant intervention effect with a medium effect size, demonstrating the HKU-strengthening effect of the developed and implemented lessons. The results are in line with other studies addressing health-related knowledge and understanding. The majority (79,4%) of the studies investigated by Demetriou et al. (26) revealed significant positive intervention effects on students' health-related fitness knowledge. Effect sizes vary from small to large, depending on the nature of moderator variables such as defined learning outcomes and implementation of PE lessons.

Due to the participatory process in our intervention, those moderator variables also varied considerably in the involved schools. Therefore, we looked at school specific differences in the development of HKU. Students of intervention schools A, B, and D improved their HKU significantly, those at school C did not. The significant HKU-improvement of students in the intervention schools A, B, and D came along with large effect sizes (*d*_A_ = 1.3 / *d*_B_ = 1.0 / *d*_D_ = 1.3). According to Hattie (60) an average increase of *d* = 0.4 (hinge point) across different subjects is regarded as a general development of students' ability over one school year whilst attending regular lessons. Thus, students at the interventions schools evidently over-performed. In contrary, students at intervention school C (*d*_C_ = 0.2) improved their HKU less than the hinge point.

Differences match with findings in relation to the evaluation of program implementation. In all schools teaching units were implemented that–besides physical aspects–at least partly also addressed psychosocial aspects of health and thereby followed the required holistic understanding of health (14, 28). However, there were considerable differences concerning other principles of fostering HKU in PE (28), such as the inclusion and empowerment of all pupils via student-centered teaching approaches (49, 60) and the delivery of health-related knowledge with experiences through physical activity (61–63). Teaching units of schools A and B were fully compliant to those principles. Teachers of school D neglected some criteria of student-centered teaching approaches. However, they also prioritized educational aspects over direct health outcomes and combined delivery of health-related knowledge with physical experiences. In contrary, teachers from school C conceptualized and implemented PE lessons that were only marginally compliant to the aforementioned pedagogical principles. They only partly included cognitive activation as a student-centered teaching approach, but they did not consider elements of reflection, relevance to everyday life or collaborative learning. Furthermore, they only marginally delivered health-related knowledge, but did not combine this knowledge with physical experiences.

In summary, a participatory planning process involving teachers, principals, students and scientists is an adequate approach to conceptualize and implement evidence-based teaching methods within the school setting. Due to this intervention, students' HKU increased. An insight, which confirms existing findings from participatory approaches, which addresses active lifestyles in PE (64) or health promotion in the whole school context (65). The present study purposefully dispensed with any structured intervention programs for teachers to follow. Instead, students as well as teachers had the chance to articulate their specific needs and interests, but also their concerns about factors hindering the implementation of planned lessons. Thereby, teachers developed an understanding of effective teachings strategies in PE and carefully reflected on their current teaching methods and the existing practices at their schools. This is regarded as an important factor influencing the contribution of staff to health education at schools (66). Additionally, the integration of students in a partnership of equals in the planning groups increased their satisfaction with the process and their motivation to learn–important effects, which already have been found in other school health promotion interventions (67). However, the participatory planning does not always work in the intended manner (see results from school C), emphasizing that numerous contextual factors influence the implementation process (68, 69). In the context of the present study, the issue of individual readiness for organizational change arose particularly (70). It turned out that the readiness of the involved stakeholders to invest personal resources to change existing processes and beliefs is crucial. In that sense, teachers of school C were not willing to reflect and change their current teaching methods.

### Strengths and Limitations

There are several strengths of this study. Through the participatory approach, we ensured that there was consideration of the students' voice and individual school requirements in health-promotion interventions within the school setting (7, 9). Furthermore, as there is a lack of an accurate empirical assessment of the outcomes of implemented teaching learning processes (23, 26), we used a well-developed and validated questionnaire for assessing the effects of our intervention on HKU. For data analysis, we used triangulation of techniques (i.e., comparison of data obtained by differing information collection techniques) and triangulation of analysis (contrasting and comparing the data analyses performed by different analysts to strengthen the credibility and confirmability of the study results) (71). However, some limitations do also exist. First, schools were not randomly assigned to either intervention or control group and the allocation was based on their willingness. However, criteria of randomization may be difficult to achieve within the school setting (5). Additionally, measurement of students' physical activity as well as school's climate, culture and ethos^4^ were not included, which could have provided further details for interpreting the results (72).

## Conclusion

This study contributes to existing research on strengthening health-related knowledge and understanding in PE. It specifically investigates a participatory approach to develop and implement evidence-based teaching units for PE. The results are encouraging, showing the potential effects of this approach, if relevant principles of fostering HKU in PE are considered.

As strengthening of HKU is an accepted demand in German as well as international PE curricula, future efforts should focus on enabling PE teachers to consistently align their teaching with those PE curricula demands. Therefore, the follow-up project Health.edu PLUS will specifically address the participatory elaboration of a variety of best-practice examples in cooperation with different stakeholders concerned. Those exemplary teaching units will specifically address how to combine the delivery of health-related knowledge with physical experiences in PE. This can help to mitigate concerns expressed by some PE teachers that an intensified delivery of health-related knowledge could significantly reduce time for movement in PE (73). The elaborated examples will be linked to an Bavarian online platform, where all interested teachers can find information about PE curricula demands and can download exemplary teaching units to fulfill those demands. The provision and dissemination of agreed-upon examples of good practice will serve as a trigger for further development and implementation of strengthening HKU in PE.

## Data Availability Statement

The datasets generated for this study are available on request to the corresponding author.

## Ethics Statement

Ethical approval was obtained from the data security official of the University of Erlangen-Nuremberg and the Bavarian State Ministry for Education and Cultural Affairs (reference number X.7-BO4106/459/8). Parents and teachers provided written informed consent; additionally, all students provided written informed consent to participate in the study as well.

## Author Contributions

HS was a major contributor in writing the manuscript and contributed to data collection. KP managed the data collection of the study and wrote a first draft of some sections of the manuscript. CT contributed to critical revision of the manuscript. RS contributed to the conception and design of this study. ST contributed to the conception and design of this study, wrote a first draft of some sections of the manuscript and contributed to critical revision of the manuscript. All authors read and approved the final manuscript.

## Conflict of Interest

The authors declare that the research was conducted in the absence of any commercial or financial relationships that could be construed as a potential conflict of interest.

## Footnotes

^1^In Germany, there are 16 state-specific curricula, since education is a matter of federal states.

^2^The project is part of a transdisciplinary research network *Capital4Health* (https://www.capital4health.de), which aims at fostering health enhancing physical activity in various population segments and settings.

^3^In Germany, there are differences between secondary modern schools and grammar schools in relation to their educational level. Secondary modern schools are for practical-minded students and prepare for vocational training. Grammar schools are attended by students with above-average abilities and motivation and lead to the exam required for studies at university/college.

^4^This is due to a very restrictive approval procedure of the Bavarian State Ministry of Education and Cultural Affairs.

## Abbreviations

HKU: Health-related knowledge and understanding
PE: Physical education
PL: Physical literacy.
